# Improving health literacy of antifungal use—Comparison of the readability of antifungal medicines information from Australia, EU, UK, and US of 16 antifungal agents across 5 classes (allylamines, azoles, echinocandins, polyenes, and others)

**DOI:** 10.1093/mmy/myad084

**Published:** 2023-08-10

**Authors:** John E Moore, Ka Wah Kelly Tang, Beverley C Millar

**Affiliations:** School of Biomedical Sciences, Ulster University, Cromore Road, Coleraine BT52 1SA, Northern Ireland, UK; Laboratory for Disinfection and Pathogen Elimination Studies, Northern Ireland Public Health Laboratory, Belfast City Hospital, Lisburn Road, Belfast BT9 7AD, Northern Ireland, UK; School of Biomedical Sciences, Ulster University, Cromore Road, Coleraine BT52 1SA, Northern Ireland, UK; School of Biomedical Sciences, Ulster University, Cromore Road, Coleraine BT52 1SA, Northern Ireland, UK; Laboratory for Disinfection and Pathogen Elimination Studies, Northern Ireland Public Health Laboratory, Belfast City Hospital, Lisburn Road, Belfast BT9 7AD, Northern Ireland, UK

**Keywords:** antifungal, fungal resistance, dermatophyte, health literacy, readability

## Abstract

Adherence to antifungals is poor in high endemic regions where antifungal resistance is high. Poor readability of prescription/over-the-counter (OTC) antifungals may contribute to poor adherence, due to the patient not fully understanding the purpose, importance, and dosage of their antifungal medicine. As there are no reports on the readability of antifungals, this study examined the readability of patient-facing antifungal information. Antifungals (*n* = 16; five classes [allylamines, azoles, echinocandins, polyenes, and others—flucytosine and griseofulvin]) were selected. Readability of four sources of information, (i) summary of product characteristics, (ii) patient information leaflets (PILs), (iii) OTC patient information, and (iv) patient web-based information, was calculated using *Readable* software, to obtain readability scores [(i) Flesch Reading Ease [FRE], (ii) Flesch–Kinkaid Grade Level [FKGL], (iii) Gunning Fog Index, and (iv) Simple Measure of Gobbledygook (SMOG) Index) and text metrics [word count, sentence count, words/sentence, and syllables/word]. PILs, web-based resources, and OTC patient information had good readability (FRE mean ± sd = 52.8 ± 6.7, 58.6 ± 6.9, and 57.3 ± 7.4, respectively), just falling short of the ≥ 60 target. For FKGL (target ≤ 8.0), PILs, web-based resources, and OTC patient information also had good readability (mean ± sd = 8.5 ± 1.0, 7.2 ± 0.86, and 7.8 ± 0.1, respectively). Improved readability scores observed correlate with reduced words, words/sentence and syllables/word. Improving readability may lead to improved patient health literacy. Healthcare professionals, academics, and publishers preparing written materials regarding antifungals for the lay/patient community are encouraged to employ readability calculators to check the readability of their work, so that the final material is within recommended readability reference parameters, to support the health literacy of their patients/readers.

Antifungal resistance has now emerged as a global concern in the successful treatment of fungal infections, including *Candida auris*.^1,2^ Patient compliance with correctly taking antifungal medication has been suggested as a driver of such resistance.^3^One aspect of the study of poor patient compliance to antifungal therapy that has not been examined to date has been how well antifungal medicines information is written for patients, parents of infected children with the responsibility of administering medicine to their child outside the hospital, as well as with carers. Antifungal Patient Information Leaflets (PILs) are enclosed with prescription drugs by the dispensing pharmacist and these are crucial in providing key information about dose, administration, side effects, and safety precautions. A previous study has indicated the importance of evaluating the readability of PILs attached to medication, as low-quality information provided could potentially lead to increased patient misuse and cause lower compliance to taking the anti-infectives correctly.^4^

The aim of this study was, therefore, to examine the readability (Flesch Reading Ease [FRE], Flesch–Kinkaid Grade Level [FKGL], Gunning Fog, and SMOG scores; text metrics) of antifungal medication aimed at (i) healthcare professionals (Summary of Product Characteristics [SPC]) and (ii) patient-facing (patient web resource, PILs, and over-the-counter [OTC] patient information), relating to antifungals (*n* = 16; five antifungal classes), from four geographical regions (Australia, EU, UK, and USA), in order to establish (i) how readable antifungal information from different article types (SPC; PILs; OTC patient information; and patient web-based information) resources from four geographical (Australia, EU, UK, and USA) regions compare to readability reference standards, (ii) if there are differences in readability between five different classes of antifungal agents (allylamines, azoles, echinocandins, polyenes, and others) examined, and (iii) if there are differences in readability between antifungal agents which are administered via different formulations (oral/intravenous [IV]/topical cream).

Antifungal agents (*n* = 16), which are used to treat human fungal infections were selected for investigation.^5^These included five classes of antifungal agents, including allylamines (terbinafine); azoles (clotrimazole, econazole, fluconazole, itraconazole, ketoconazole, miconazole, posaconazole, and voriconazole); echinocandins (anidulafungin, caspofungin, and micafungin); polyenes (amphotericin B and nystatin); and others (flucytosine and griseofulvin). Four sources of information on antifungal medicines, aimed at healthcare professionals, patients, and the general public, were obtained from publically and freely available web resources, including (i) Summaries of Product Characteristisc (SPCs) (*n* = 30) were sourced from the Australian Medicines regulator, the Therapeutic Goods Administrator (TGA) through the Australian Register of Therapeutic Goods (ARTG) (https://compliance.health.gov.au/artg/), (ii) US Medication Guidance (www.drugs.com) (*n* = 30), (iii) OTC antifungal patient information (*n* = 30) from UK high street and UK online pharmacies, and (iv) PILs (*n* = 31) from the European Medicines Agency (EMA) (https://www.ema.europa.eu/en).

Each source of antifungal information was examined using the online subscription-based software tool, *Readable* (www.readable.com), which was used in accordance with the website's instructions. The software was used to calculate four readability scores, including (i) FRE, (ii) FKGL, (iii) Gunning Fog Index, and (iv) SMOG Index, as detailed in Supplementary File 1. An additional four text metrics were also calculated, including word count, sentence count, words per sentence, and syllables per word. These readability measures were chosen for examination as most readability studies frequently employ and quote the results of these. Readable.com was selected as the preferred online calculator, as it has been previously used in several healthcare readability studies,^6,7^ as well as in a recent study which compared a variety of online readability calculators and concluded that *Readable* was the optimum calculator to use due to its accuracy, user experience, and capacity to examine multiple readability parameters from clinical materials.^8^

The readability data obtained underwent statistical analyses using GraphPad PRISM version 9 (Boston, USA). To determine if the data followed a normal distribution, a normality test was performed on each set of data using the Kolmogorov–Smirnov test. Dependent on the normality of data distribution, for data that were normally distributed, one-way Anova (parametric) was performed, with a posthoc Tukey multiple comparisons test, to compare the means of normally distributed parameters. Datasets that were not normally distributed, the Kruskal–Wallis (non-parametric) test with Dunn's adjusted *P* values was performed. A *P* value of < .05 (5%) was considered as statistically significant.

A total of 121 sources of antifungal medicine information were analysed. Readability scores for the FRE and the FKGL, for each antifungal information type, are shown in Fig. 1a,b, respectively. Text metrics, including words per sentence and syllables per word, for each antifungal information type, are shown in Fig. 1c,d, respectively. The Gunning Fog score and the SMOG score for each antifungal information type are shown in Supplementary File 2 and additional text metrics, including word count and sentence count, are shown in Supplementary File 3. Readability analyses of antifungal information, based on antifungal class (Fig. 2a,b) and formulation/route of administration (oral versus/iv/topical cream) is shown (Fig. 2c,d).

**Figure 1.**
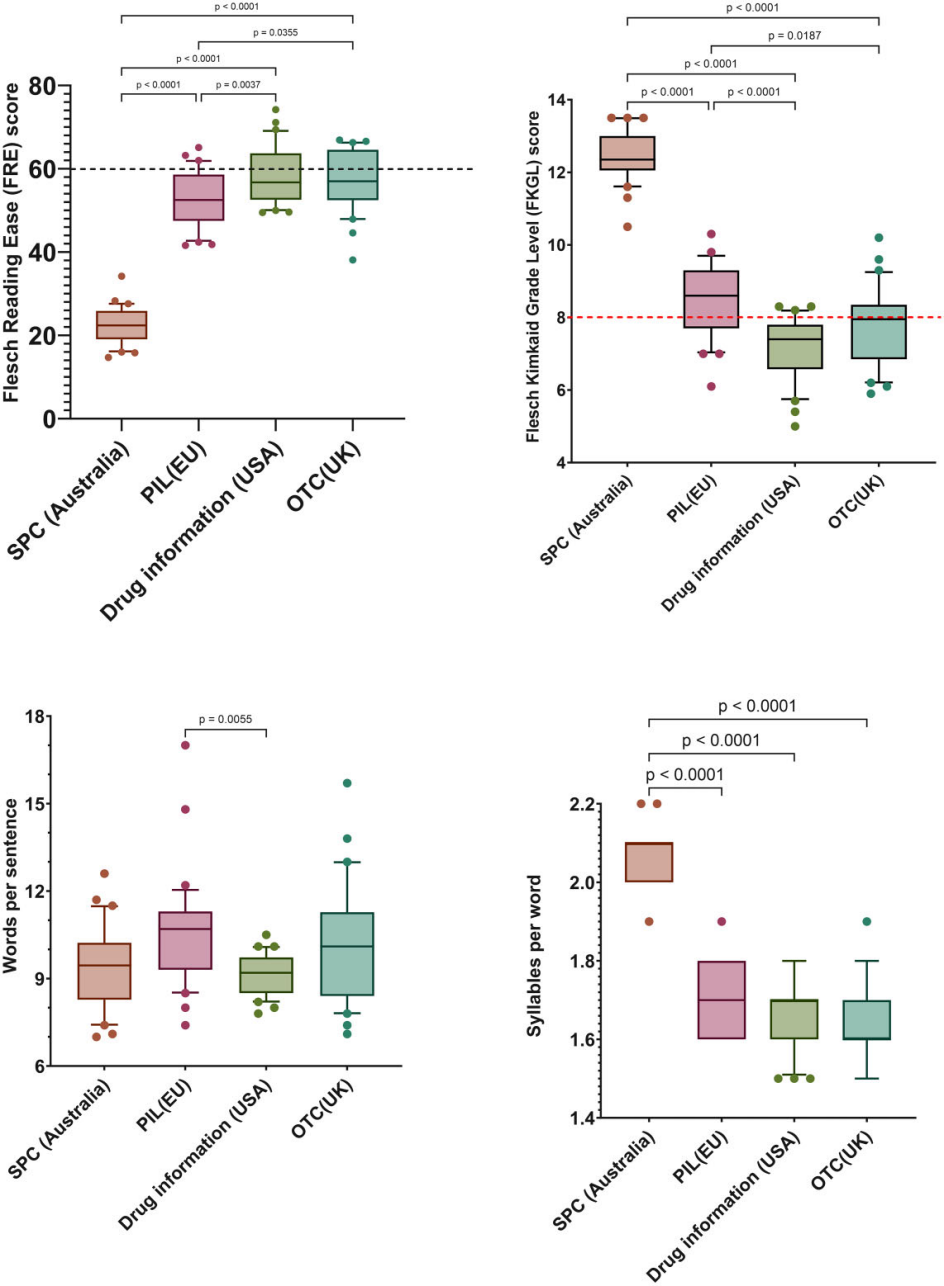
Box and whisker plot comparing readability scores for (A) Flesch Reading Ease (FRE); (B) Flesch–Kincaid Grade Level (FKGL); and text metric scores, (C) words per sentence and (D) syllables per word. These were calculated from (i) Summary of Product Characteristics (SPC), Source: Australian Register of Therapeutic Goods (ARTG) (*n* = 30); (ii) US Medication Guidance, Source: www.drugs.com (*n* = 30); (iii) Over-The-Counter (OTC) antifungals, Source: Patient information from UK High Street and online pharmacies (*n* = 30), and (iv) EU Patient Information Leaflets (PILs), Source :European Medicines Agency (*n* = 31). The box represents 25^th^ and 75^th^ percentile and the bar represents the median. Whiskers represent the 10^th^ and 90^th^ percentile and ● represent outliers outside these percentile ranges. For (A) FRE and (B) FKGL, statistical significance is shown, calculated using Anova with a posthoc Tukey's multiple comparisons test (parametric). For (C) Words per sentence and (D) Syllables per word, statistical significance is shown, calculated using the Kruskal–Wallis (non-parametric) test with Dunn's Adjusted p values. A *P* value of < .05 (5%) was considered as statistically significant. The dashed red line represents the target readability score. For the FRE, this is ${\ge}$ 60. For the FKGL score, this is ≤ 8.

**Figure 2.**
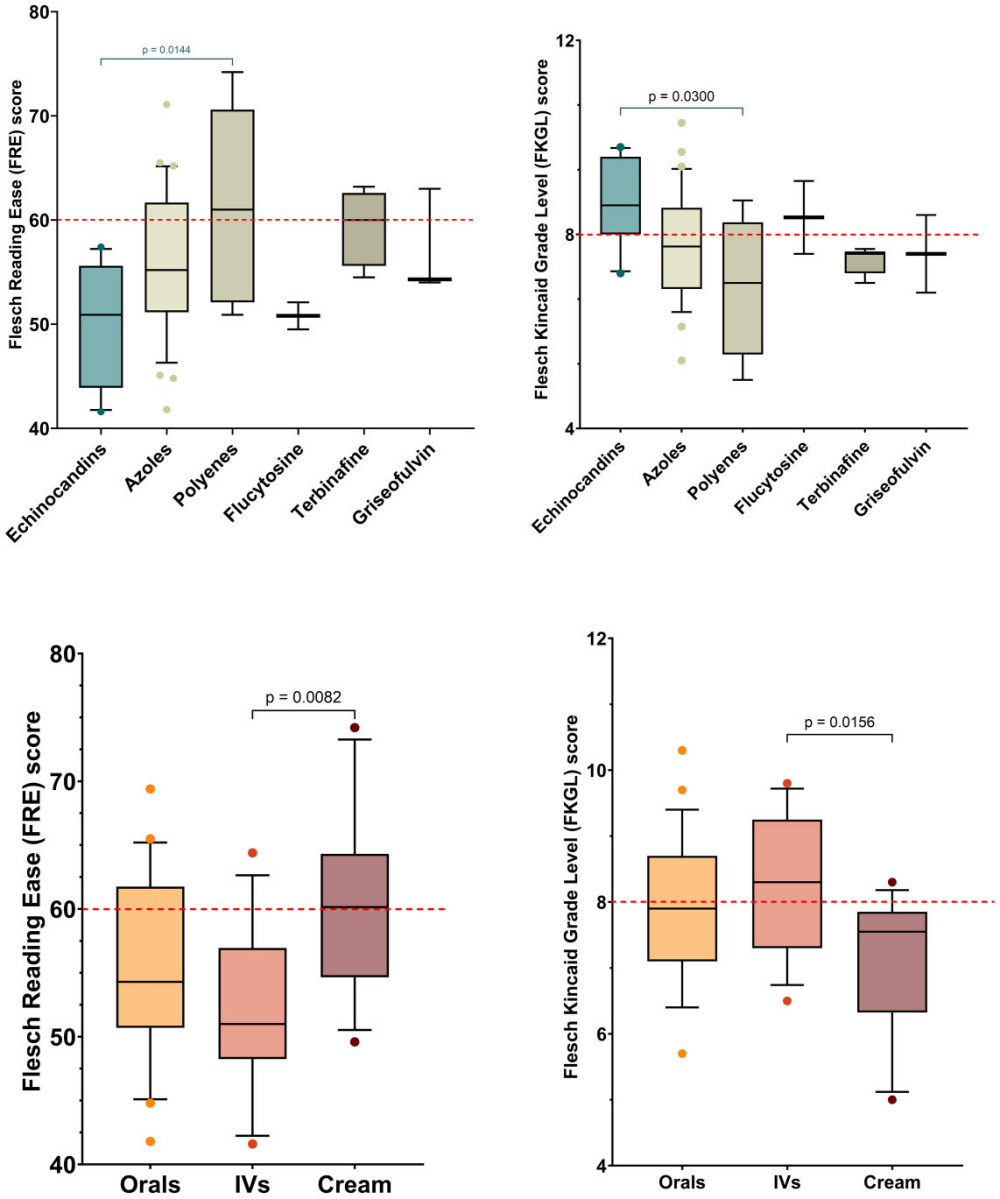
Box and whisker plot comparing (A) the Flesch Reading Ease (FRE) score and (B) the Flesch–Kincaid Grade Level (FKGL) for five classes of antifungal medicines (allylamines [terbinafine]; azoles [clotrimazole, econazole, fluconazole, itraconazole, ketoconazole, miconazole, posaconazole, and voriconazole]; echinocandins [anidulafungin, caspofungin, and micafungin]; polyenes [amphotericin B and nystatin]; and others [flucytosine and griseofulvin]). Antifungal information was obtained from patient-facing sources i.e., the US Medication Guidance, Source: www.drugs.com (*n* = 30) and (ii) EU Patient Information Leaflets (PILs), Source :European Medicines Agency (*n* = 31). The box represents 25^th^ and 75^th^ percentile and the bar represents the median. The dashed red line represents the target readability score (FRE $\ge$60; FKGR ≤ 8). Whiskers represent the 10^th^ and 90^th^ percentile and ● represent outliers outside these percentile ranges. Statistical significance is shown, calculated using Anova with a posthoc Tukey's multiple comparisons test (parametric). A *P* value of < .05 (5%) was considered as statistically significant. The box and whiskers plot comparing (C) the FRE score and (D) the FKGL for three formulations/routes of medicine administration (*n* = 58) comprising of orals (*n* = 29), iv (*n* = 17), and topical creams (*n* = 12). Antifungal information was obtained from patient-facing sources i.e., the US Medication Guidance, Source: www.drugs.com (*n* = 27) and (ii) EU PILs, Source :European Medicines Agency (*n* = 31). The box represents 25^th^ and 75^th^ percentile and the bar represents the median. The dashed red line represents the target readability score (FRE $\ge$60; FKGR ≤ 8). Whiskers represent the 10^th^ and 90^th^ percentile and ● represent outliers outside these percentile ranges. Statistical significance is shown, calculated using Anova with a posthoc Tukey's multiple comparisons test (parametric). A *P* value of < .05 (5%) was considered as statistically significant.

To our knowledge, this is the first study to conduct an assessment of the readability of antifungal agents employed in the treatment of fungal infections. Readability has become a commonly employed tool to help healthcare professionals prepare patient-facing materials and resources, supported by a growing evidence-base of published literature. In this study, we examined the readability of antifungal information gathered from four sources, including one healthcare professional-facing and three patient-facing sources. Recommendations for suitable readability levels can vary between institutions, with the American Medical Association recommending that all patient-facing material be written at a sixth-grade level (11 years old).^9^ Conversely, the Centers for Disease Control and Prevention (CDC) recommends that patient-facing information does not surpass an eighth-grade reading level (13 years old).^10^ A FRE score of ≥ 60 is the target for this score. Overall, our data suggests that antifungal information from patient-facing information, including EU PILs, US web-based resources, and UK OTC patient information had good readability (FRE [mean ± sd] 52.8 ± 6.7, 58.6 ± 6.9, and 57.3 ± 7.4, respectively), just falling short of the ≥ 60 target. Likewise, with the FKGL (target ≤ 8.0), the PILs, web-based resources, and OTC patient information had good readability (8.5 ± 1.0, 7.2 ± 0.86, and 7.8 ± 0.1, respectively), where both the web-based resources and OTC patient information achieved the target FKGL readability target of ≤ 8.0. In addition, one of these sources, namely the SPC, which is aimed at healthcare professionals, unsurprisingly had the poorest readability of all the information sources examined (FRE mean = 22.5 ± 4.4 [sd]; FKGL mean = 12.4 ± 0.7 [sd]). This information, whilst in the public domain, would not be easily read by patients and the general public and therefore should not be the sole source of antifungal information provision for patients. We further examined the readability of antifungal patient-facing information, by antifungal class. Mean FRE scores were best for polyenes (61.5 ± 9.8 [sd]), terbinafine (59.3 ± 3.6), griseofulvin (57.1 ± 5.1), azoles (56.1 ± 7.0), flucytosine (50.8 ± 1.8), and lastly echinocandins (49.8 ± 5.5). A similar result was seen with mean FKGL scores, with the azoles, polyenes, terbinafine, and griseofulvin < 8.0 (target FKGL), with the other classes (echinocandins and flucytosine) slightly over the target value. When examined by formulation, topical cream formulations gave a mean FRE score of 60.4 ± 7.2, followed by the orals at 55.3 ± 7.0. IVs had a mean FRE score of 52.2 ± 6.7. Readability of those antifungal formulations, i.e., oral and creams, where patients have autonomy relating to their compliance in the community need to have optimal readability scores, so that their reading of these at home is not impeded by poor clarity and difficulty in reading such information, in order to encourage and support optimal compliance.

Patient-facing antifungal information from three sources (PILs, web-based information, and OTC information), whilst not absolutely meeting suggested readability criteria, was highly readable. We, therefore, advocate that authors of patient-facing antifungal information are aware of the utility of readability tools to enable authors to write information on antifungal medicines, with further enhanced readability for patients and the public.

## Supplementary Material

myad084_Supplemental_Files

## References

[1] Chakrabarti A, Singh S. Multidrug-resistant *Candida auris*: An epidemiological review. Expert Rev Anti Infect Ther. 2020; 18: 551–562. 32237924 10.1080/14787210.2020.1750368

[2] Gupta AK, Renaud HJ, Quinlan EM, Shear NH, Piguet V. The growing problem of antifungal rresistance in onychomycosis and other superficial mycoses. Am J Clin Dermatol. 2021; 22: 149–157. 33354740 10.1007/s40257-020-00580-6

[3] Saunte DML, Pereiro-Ferreirós M, Rodríguez-Cerdeira C et al. Emerging antifungal treatment failure of dermatophytosis in Europe: Take care or it may become endemic. J Eur Acad Dermatol Venereol. 2021; 35: 1582–1586. 33768571 10.1111/jdv.17241

[4] Munsour EE, Awaisu A, Hassali MA et al. Readability and comprehensibility of patient information leaflets for antidiabetic medications in Qatar. J Pharm Technol. 2017; 33: 128–136. 34860991 10.1177/8755122517706978 PMC5998532

[5] Chen SC, Sorrell TC. Antifungal agents. Med J Aust. 2007; 187:404–409. 17908006 10.5694/j.1326-5377.2007.tb01313.x

[6] Meleo-Erwin Z, Basch C, Fera J et al. Readability of online patient-based information on bariatric surgery. Health Promot Perspect. 2019; 9: 156–160. 31249804 10.15171/hpp.2019.22 PMC6588814

[7] Patel PA, Gopali R, Reddy A et al. The readability of ophthalmological patient education materials provided by major academic hospitals. Semin Ophthalmol. 2022; 37: 71–76. 33852375 10.1080/08820538.2021.1915341

[8] McGrath L, Millar BC, Moore JE. Using plain language to communicate with clinical trials participants: Comparison of readability calculators. Contemp Clin Trials. 2022; 123:106995. 36347454 10.1016/j.cct.2022.106995

[9] Weiss BD . Health literacy: A Manual for Clinicians. Chicago, IL: American Medical Association Foundation and American Medical Association, 2003.

[10] Kecojevic A, Basch CH, Garcia P. Readability analysis of online health information on preexposure prophylaxis (PrEP). Public Health. 2020; 182: 53–55. 32171091 10.1016/j.puhe.2020.02.002

