# Supplementary figures and images for: Improving health literacy of antifungal use—Comparison of the readability of antifungal medicines information from Australia, EU, UK, and US of 16 antifungal agents across 5 classes (allylamines, azoles, echinocandins, polyenes, and others)
Source: Med Mycol. 2023 Aug 10;61(9):myad084. doi: 10.1093/mmy/myad084 (PMC10802897; doi:10.1093/mmy/myad084)

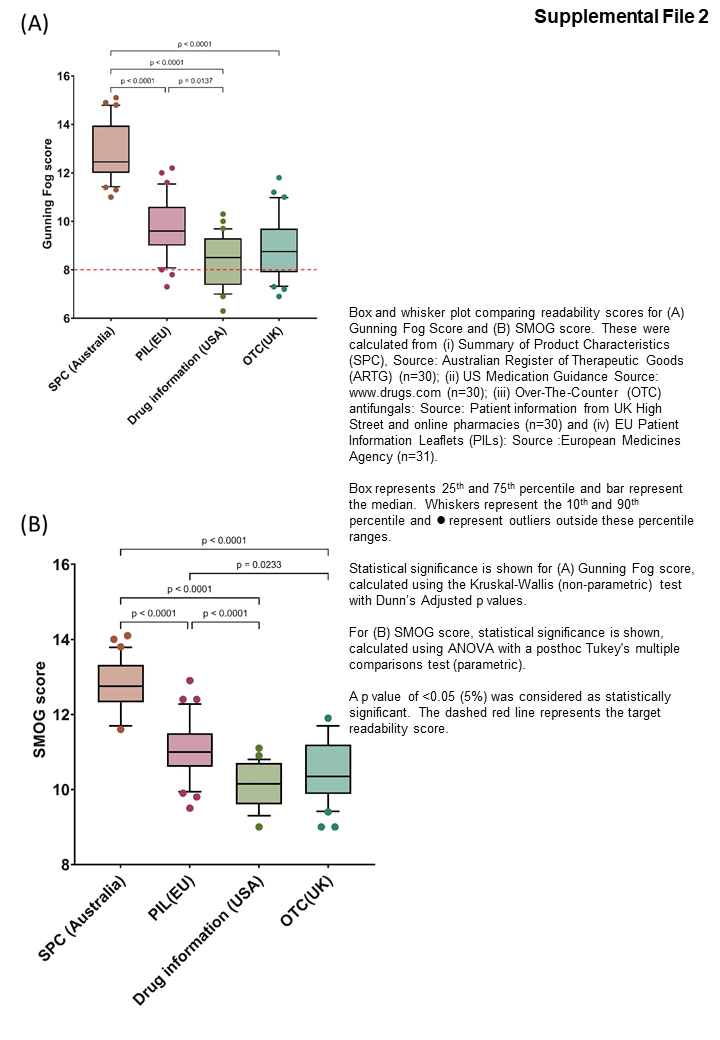

Supplement: myad084_Supplemental_Files [file myad084_supplemental_files.zip › mm-2023-0154-File009.tif]

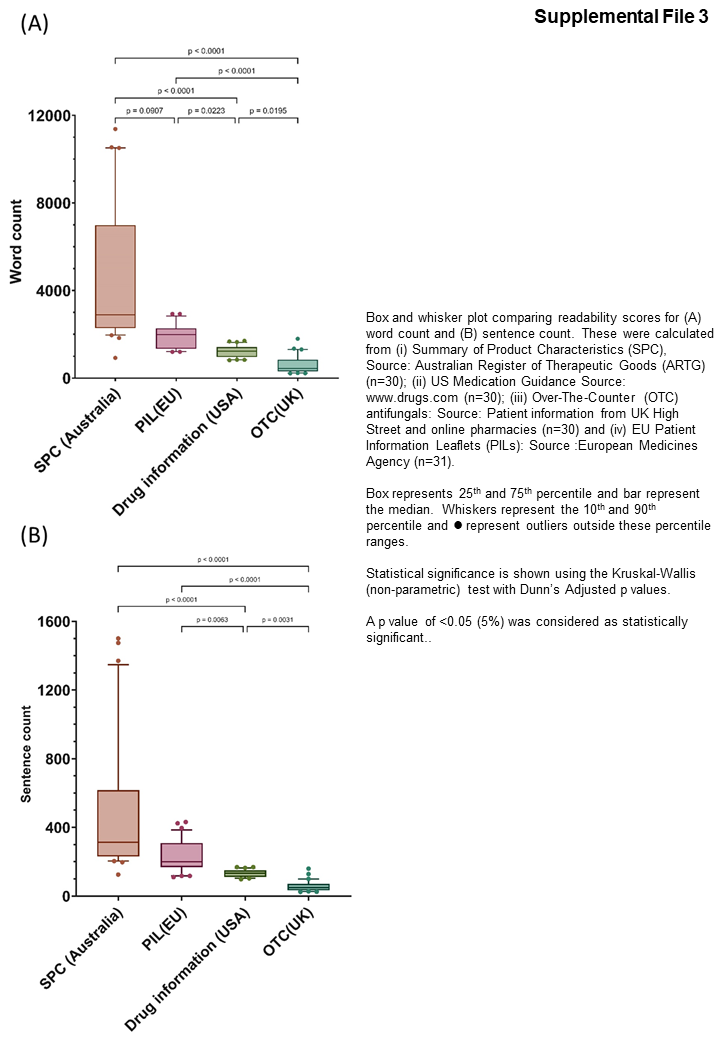

Supplement: myad084_Supplemental_Files [file myad084_supplemental_files.zip › mm-2023-0154-File010.tif]

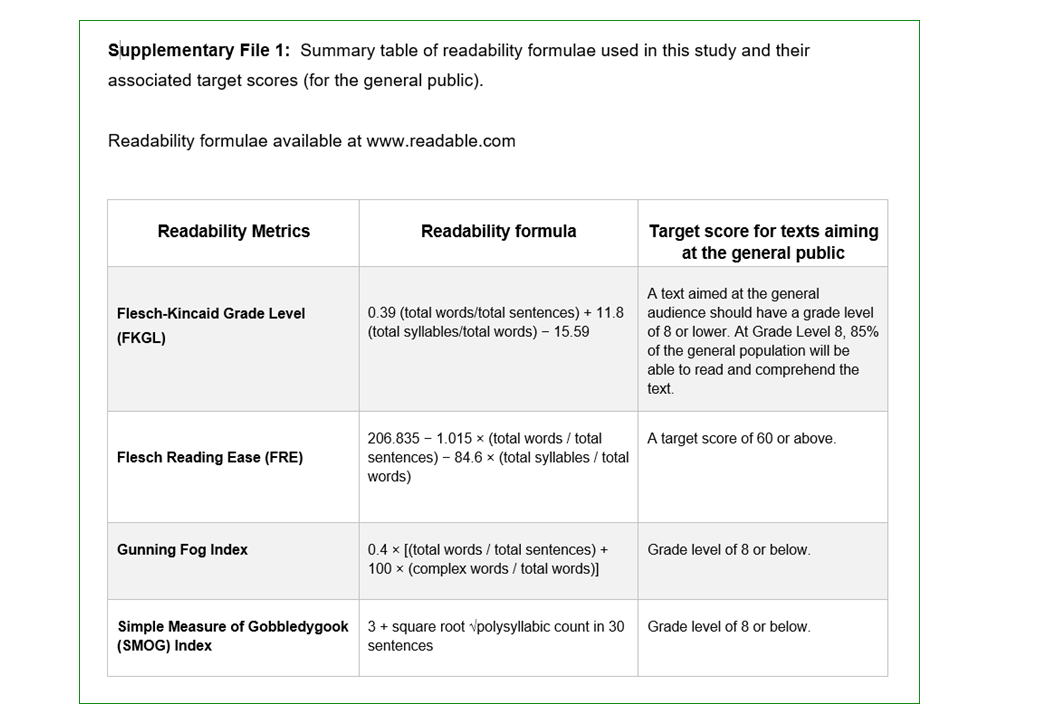

Supplement: myad084_Supplemental_Files [file myad084_supplemental_files.zip › mm-2023-0154-File008.tif]
